# Lumbar hernia diagnosed after laparoscopic hiatal hernia surgery

**DOI:** 10.1002/ccr3.3795

**Published:** 2021-01-19

**Authors:** Jian Shen Kiam, Stephen Lam, Jasmine Crane, Bhaskar Kumar

**Affiliations:** ^1^ Norfolk and Norwich University Hospitals NHS Foundation Trust Norfolk UK; ^2^ University of East Anglia Medical School Norfolk UK

**Keywords:** laparoscopic, lumbar hernia, superior lumbar triangle, surgery

## Abstract

The presence of a new lumbar swelling or pain in the postoperative period following laparoscopic surgery should raise the suspicion of a lumbar hernia. Cross‐sectional imaging can be used to establish an early diagnosis to enable successful management.

## Graphical abstract

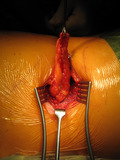

## 1 INTRODUCTION

Lumbar hernias occur through defects in the lumbar muscles or the posterior fascia, below the 12th rib, accounting for less than 2% of all abdominal hernias.^1^ In 1886 and 1870, respectively, Grynfeltt and Lesshaft describe hernias which specifically occur through the superior lumbar triangle known as the Grynfeltt‐Lesshaft triangle; a space bounded medially by the quadratus lumborum muscle, superiorly by the twelfth rib, and laterally by the internal oblique muscle.^2^, ^3^

We report a case of a patient who presented with a hernia through the superior lumbar triangle, following laparoscopic paraesophageal hernia repair four months previously. This case suggests pneumoperitoneum in laparoscopic surgery may be a potential cause of lumbar hernia and identifies important considerations when patients present with lumbar swellings and obstructive symptoms postoperatively.

## 2 CASE HISTORY

A 66‐year‐old woman presented to hospital with pain over her right lumbar region associated with nausea, vomiting, and an inability to maintain oral intake. Four months previously, she had undergone laparoscopic repair of a giant hiatal hernia. The hernia sac contained most of the stomach and omentum, consistent with a type III paraesophageal hernia. The patient's previous history also included a hysterectomy, osteoarthritis and a preoperative chest XR showed bilateral calcified pleural plaques consistent with previous asbestos exposure. There was no past medical history of other respiratory disease or reduced respiratory function. On examination a tender right lumbar swelling approximately 3x5cm was noted. The patient's blood pressure, heart rate, temperature, oxygen saturations on air and respiratory rate were all within normal range. Her CT scans before her hiatal hernia surgery did not show any evidence of a lumbar hernia.

### 2.1 Investigations

Laboratory blood tests for full blood count, C‐reactive protein, liver, and renal function were within normal limits.

**FIGURE 1:**
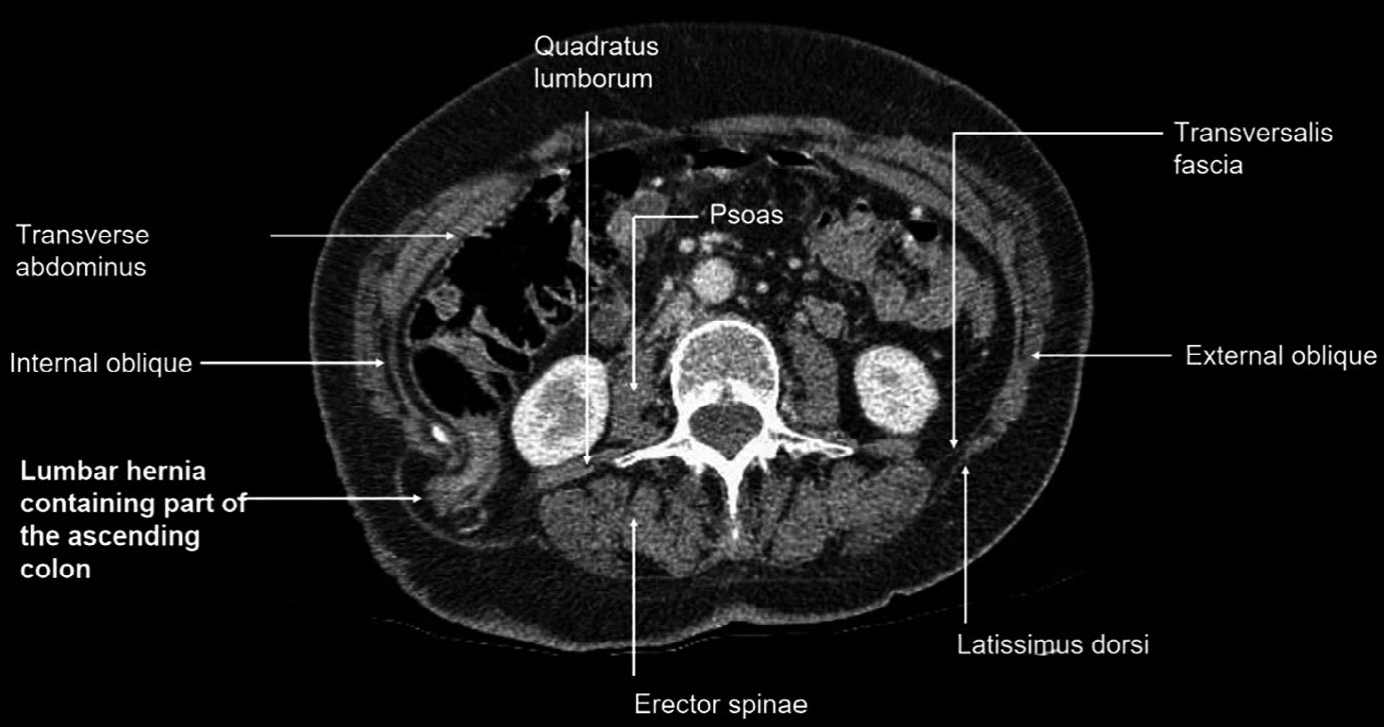
Transverse slice of CT abdomen showing ascending colon in hernial sac

**FIGURE 2:**
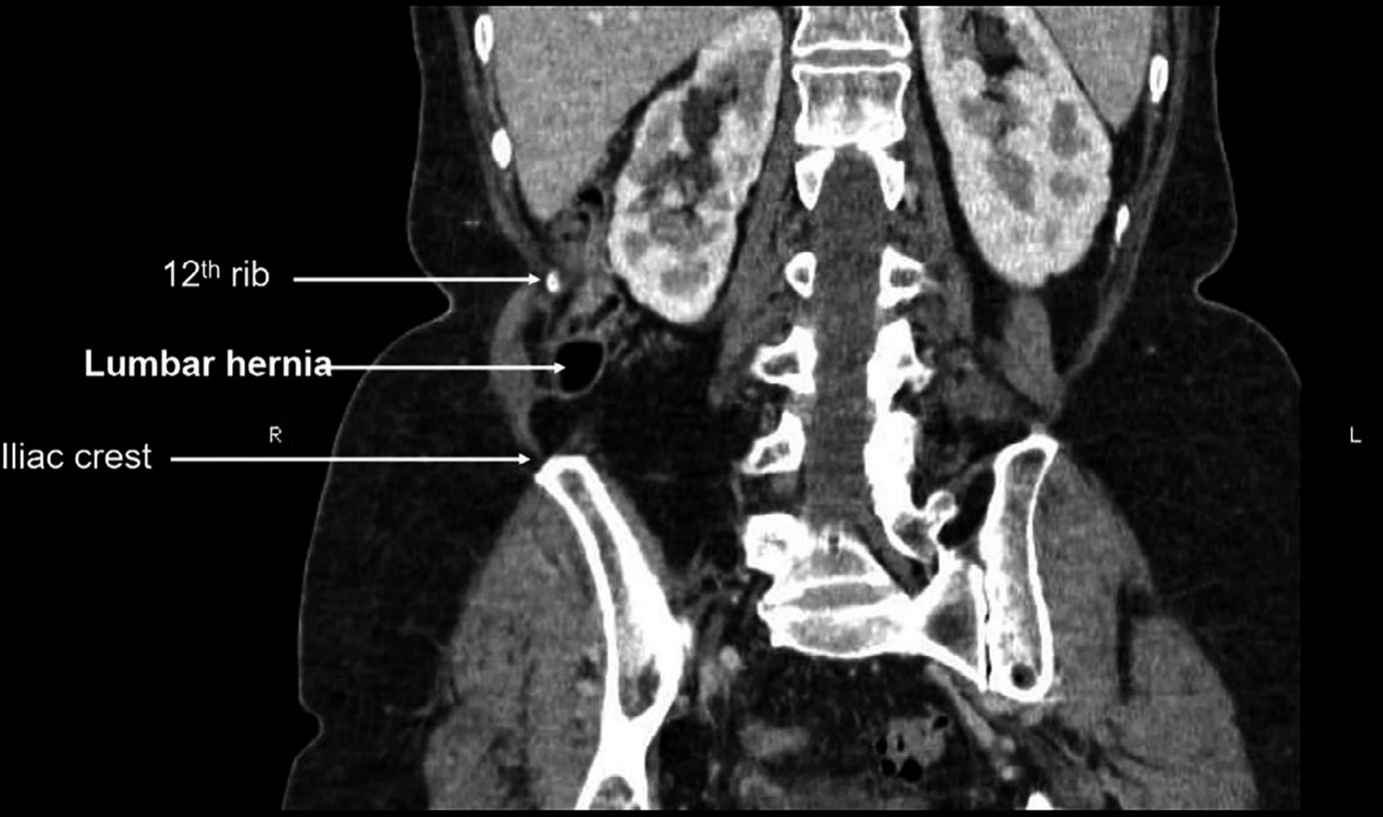
Coronal slice of CT abdomen showing area of hernia relative to 12th rib and iliac crest

A computed tomography (CT) scan reported a 38 x 81 millimeter right paralumbar hernia containing fat and part of the ascending colon. The hernia neck measured 26mm. The upstream ascending colon was prominent suggesting a degree of obstruction (Figures 1 and 2).

### 2.2 Treatment

**FIGURE 3:**
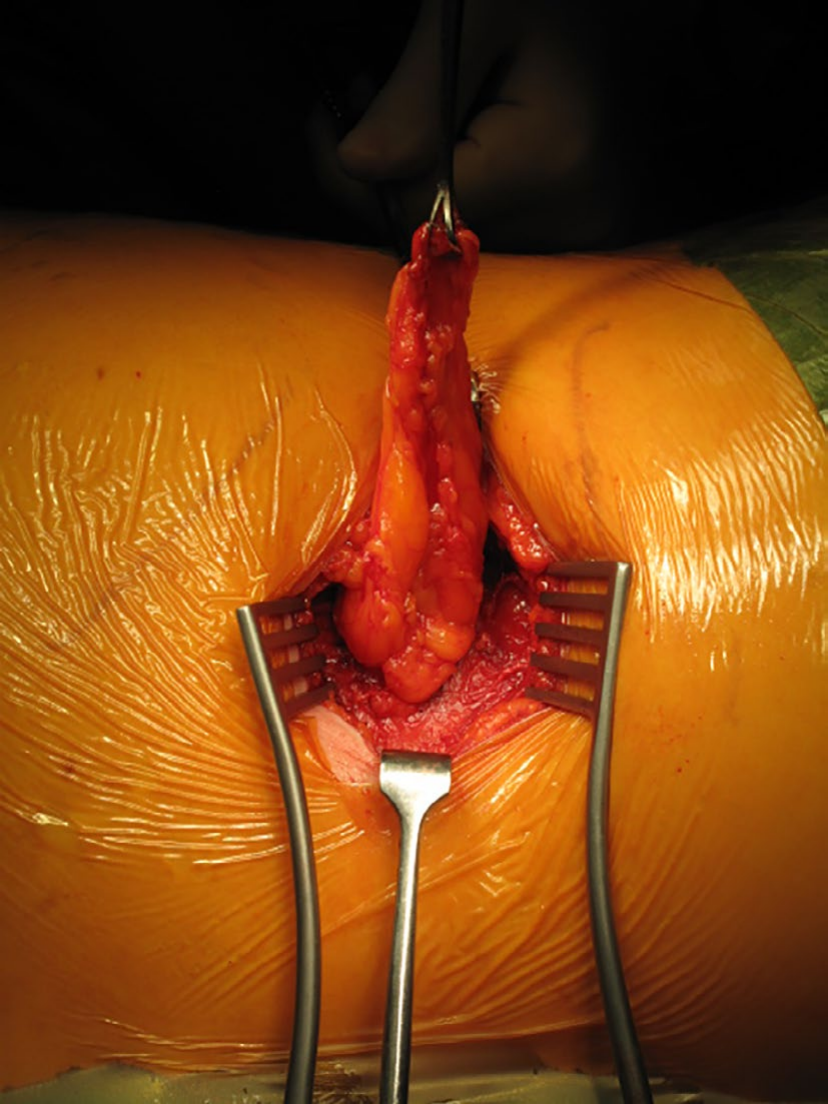
Visualization of the lumbar hernia

**FIGURE 4:**
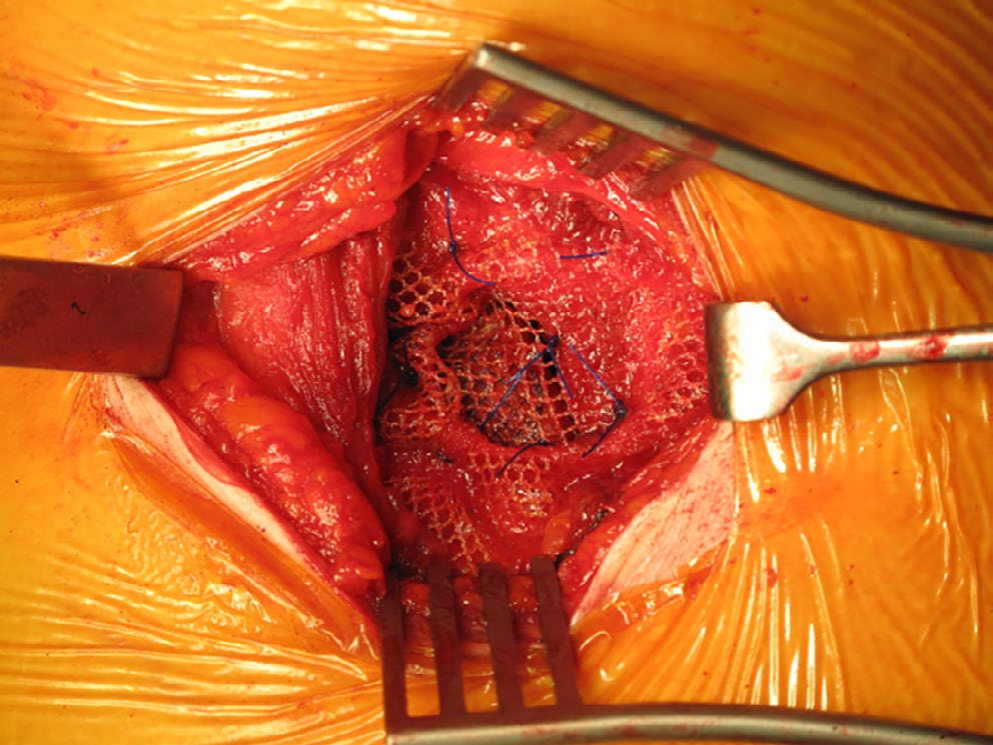
Insertion of mesh secured with sutures in a sublay position

The patient underwent open emergency repair of the hernia using a transverse right lumbar incision. She was found to have a 3 ‐ 4cm hernia defect through the superior lumbar triangle (Grynfeltt‐Lesshaft triangle) containing a large amount of extraperitoneal fat but no bowel (Figure 3). The hernia contents were reduced easily and an ULTRAPRO Hernia System® (Monocryl® and Prolene® filament construction mesh) was placed and secured with 2.0 Prolene® sutures into a sublay position (Figure 4). The patient made a full recovery postoperatively and was discharged the following day.

### 2.3 Outcome and Follow‐up

The patient was readmitted day 14 postoperatively with a superficial hematoma requiring bedside drainage. At 6‐month follow‐up, the patient was well with no further postoperative complications.

## 3 DISCUSSION

First described in 1672, lumbar hernias can be of three described types: superior lumbar hernia, inferior lumbar hernia, and diffuse lumbar hernia. These may herniate through two possible anatomical areas. The lumbar region is bordered superiorly by the 12th rib, inferiorly by the iliac crest, and medially by the erector spinae muscles. This area contains 2 areas of weakness: the inferior lumbar triangle and superior lumbar triangle. The superior lumbar triangle is larger of the two and is the most common location for spontaneous hernias.^4^ Described in 1866 by Grynfeltt and later in 1870 by Lesshaft, it is a space which is bounded medially by the quadratus lumborum muscle, superiorly by the twelfth rib, and laterally by the internal oblique muscle.^2^, ^3^

20% of lumbar hernias are congenital, and the remaining 80% are primary (spontaneous) or secondary (acquired).^5^ Risk factors include conditions that elevate intra‐abdominal pressure as well as aging, muscle atrophy, and extreme loss of fatty tissue.^4^ Secondary lumbar hernias can occur due to trauma, infection, or postoperative complications.^4^

Lumbar hernias are difficult to diagnose as they often present asymptomatically or with nonspecific symptoms such as back pain. They most commonly present as a posterolateral mass that is usually reducible increasing with a rise in intra‐abdominal pressure and reducing when the patient assumes the prone position.^5^ Approximately, 9% of patients presenting with lumbar hernia have associated bowel obstruction.^4^ Low suspicion can lead to misdiagnosis of other pathologies such as lipomas or retroperitoneal tumors. Therefore, in the presence of suggestive symptoms, lumbar hernias should remain a differential especially if there are risk factors.

Our patient did not report having any mass or lumps prior to her initial laparoscopic paraesophageal hernia repair. Symptoms in relation to this hernia were evident several weeks after the initial laparoscopic hiatal hernia repair.

Although lumbar hernias have been reported following open surgery, it has not been reported following laparoscopic surgery. Given the chronology of events in this case, it may be postulated that the raised intra‐abdominal pressure of laparoscopic surgery may have exacerbated a small pre‐existing asymptomatic lumbar hernia.

To our knowledge, there are no reported cases in the literature linking lumbar hernias as a complication of laparoscopic surgery.

80% of lumbar hernias as primary or secondary, within that approximately 25% of these cases are secondary to trauma or iatrogenic.^6^ There have been multiple cases of lumbar hernia occurring after an operation, these include total hip arthroplasty,^7^ as a complication secondary to iliac bone‐graft donor site,^8^ autologous latissimus dorsi breast reconstruction,^9^ and open nephrectomies.^10^ Additionally, the study looked at comparing those who developed lumbar hernias post–open nephrectomy found there was significant difference in age, operative time, presence of chronic obstructive pulmonary disease, and severe obesity (BMI > 35).^10^

In summary, if a patient presents with a new lumbar swelling in the postoperative period following laparoscopic surgery then the possibility of a lumbar hernia needs to be considered. These hernias should thereafter be repaired to prevent acute complications such as obstruction.

## CONFLICT OF INTEREST

All authors have disclosed no conflict of interest.

## AUTHOR CONTRIBUTIONS

Jian Shen Kiam: drafted the manuscript and contributed to the study, critically revised the manuscript, and approved the final version for publication. Stephen Lam and Bhaskar Kumar: contributed to the concept and design of the study, acquisition of figures, and critical revision of the manuscript and approved the final version. Jasmine Crane: contributed to the critical revision of the manuscript and approved the final version.

## ETHICAL APPROVAL

Ethical approval was not required for this study.

## Data Availability

Data not available due to ethical and legal restrictions.
